# Meta-learning-based Inductive logistic matrix completion for prediction of kinase inhibitors

**DOI:** 10.1186/s13321-024-00838-9

**Published:** 2024-04-16

**Authors:** Ming Du, XingRan Xie, Jing Luo, Jin Li

**Affiliations:** 1 School of Software, Yunnan University, Kunming, 650091 China; 2 The Key Laboratory of Software Engineering of Yunnan Province, Kunming, 650091 China; 3 The Cloud Computing Engineering Research Center of Yunnan Province, Kunming, 650091 China; 4 State Key Laboratory for Conservation and Utilization of Bio-Resource, School of Ecology and Environment and School of Life Sciences, Yunnan University, Kunming, 650091 Yunnan China

**Keywords:** Protein kinases, kinase inhibitors, Meta-learning, Inductive logistic matrix completion

## Abstract

**Abstract:**

Protein kinases become an important source of potential drug targets. Developing new, efficient, and safe small-molecule kinase inhibitors has become an important topic in the field of drug research and development. In contrast with traditional wet experiments which are time-consuming and expensive, machine learning-based approaches for predicting small molecule inhibitors for protein kinases are time-saving and cost-effective, which are highly desired for us. However, the issue of sample scarcity (known active and inactive compounds are usually limited for most kinases) poses a challenge to the research and development of machine learning-based kinase inhibitors' active prediction methods. To alleviate the data scarcity problem in the prediction of kinase inhibitors, in this study, we present a novel Meta-learning-based inductive logistic matrix completion method for the Prediction of Kinase Inhibitors (MetaILMC). MetaILMC adopts a meta-learning framework to learn a well-generalized model from tasks with sufficient samples, which can fast adapt to new tasks with limited samples. As MetaILMC allows the effective transfer of the prior knowledge learned from kinases with sufficient samples to kinases with a small number of samples, the proposed model can produce accurate predictions for kinases with limited data. Experimental results show that MetaILMC has excellent performance for prediction tasks of kinases with few-shot samples and is significantly superior to the state-of-the-art multi-task learning in terms of AUC, AUPR, etc., various performance metrics. Case studies also provided for two drugs to predict Kinase Inhibitory scores, further validating the proposed method's effectiveness and feasibility.

**Scientific contribution:**

Considering the potential correlation between activity prediction tasks for different kinases, we propose a novel meta learning algorithm MetaILMC, which learns a prior of strong generalization capacity during meta-training from the tasks with sufficient training samples, such that it can be easily and quickly adapted to the new tasks of the kinase with scarce data during meta-testing. Thus, MetaILMC can effectively alleviate the data scarcity problem in the prediction of kinase inhibitors.

**Supplementary Information:**

The online version contains supplementary material available at 10.1186/s13321-024-00838-9.

## Introduction

The dysregulation of protein kinases plays critical roles in numerous human diseases, including cancers, inflammatory diseases, central nervous system disorders, cardiovascular diseases, and complications of diabetes, therefore protein kinases become an important source of potential drug targets [1]. At present, 71 small molecule kinase inhibitors (SMKI) have been approved by the US Food and Drug Administration (FDA), approximately half of which were approved in the past 5 years. More than 250 kinase inhibitors are in preclinical and clinical trials [2, 3]. According to SMKI clinical trial data, about 110 new kinases are currently being explored as drug targets, while about 45 targets of approved kinase inhibitors account for only about 30% of the human kinase group, indicating that small molecule kinase inhibitors still have great drug research and development value [2, 3]. Especially in the field of anti-tumor drug research and development, multitarget kinase inhibitors and highly selective kinase inhibitors can be used to treat cancer. Multiple kinase inhibitors can target a wide range of human kinases at the same time to play their anti-cancer role [4, 5]. Therefore, to fully understand and discover the potential small molecule compounds in the human Kinome, and to develop new, efficient, and safe small molecule kinase inhibitors has become an important topic in the field of drug research and development [6].

The traditional kinase inhibitors are found by low-throughput methods [7–9], that is, screening by determining the ability of compounds to reduce kinase phosphorylation activity (IC50) [10] or their binding affinity with kinases [11]. However, this method cannot be used to determine the inhibition ability of compounds to the whole Kinome. With the development of technology, it is possible to screen new high-throughput kinase profiles [12–17]. However, the long experimental cycle, high equipment requirements, and high cost make it difficult to use it as an early screen approach for drug discovery [18].

In recent years, the existing methods have accumulated a large amount of experimental data, which makes it possible to use data-driven methods to train machine learning models to predict kinase inhibitors. Compared with traditional experimental methods, machine learning methods have low experiment costs, and high efficiency, and can effectively narrow the scope of experiments and reduce experimental blindness [19]. It can be seen that the prediction method of kinase inhibitor activity based on statistical machine learning has actively promoted the development of kinase inhibitors [18–25]. Generally, there are two categories of machine learning-based approaches for finding kinase inhibitors, i.e., single kinase prediction model (SKM) and multiple kinases prediction model (MKM) [20].

### The SKM approaches

These models were separately trained with individual data sets relating to a kinase and then made predictions for the kinase. For example, Bora et al. [21] developed two-dimensional pharmacophore-based random forest models for the effective profiling of kinase inhibitors where one hundred-seven prediction models were developed to address distinct kinases spanning over all kinase groups. Merget et al. [18] presented ligand-based activity prediction models for over 280 kinases by employing Random Forest on an extensive data set of proprietary bio-activity data. The existing SKM approaches usually use statistical machine learning methods such as Naive Bayesian, random forest, etc. to build prediction models, and generally use pharmacophore fingerprints or ECFP fingerprints as compound descriptors. The experimental results of these methods show that SKM can achieve good prediction results for kinases with many known active, and inactive compounds. However, the known active, and inactive compounds of most kinases are very few. When SKM meets kinases with few samples, it always shows unsatisfactory predictive power and a tendency toward overfitting.

### The MKM approaches

These models refer to using one model to predict multiple compounds on multiple kinases (Kinome) activity at the same time. These models usually encode the kinase target, to achieve the prediction of DTI or affinity. Niijima et al. [22] proposed a de-convolution approach to dissecting kinase profiling data to gain knowledge about the cross-reactivity of inhibitors from large-scale profiling data. This approach not only enables activity predictions of given compounds on a Kinome-wide scale but also allows extraction of residue-–fragment pairs that are associated with an activity. Janssen et al. [19] presented Drug Discovery Maps (DDM) that map the activity profile of compounds across an entire protein family. DDM is based on the t-distributed stochastic neighbor embedding (t-SNE) algorithm to generate a visualization of molecular and biological similarity and maps chemical and target space to predict the activities of novel kinase inhibitors. Raquel Rodríguez-Pérez et al. [23] proposed a multi-task learning model to predict highly potent and weakly potent protein kinase inhibitors. A total of 19 030 inhibitors with activity against 103 human kinases were used for modeling. Experimental results show that multi-task learning consistently outperformed single-task modeling. Lo et al. [24] used structured domain knowledge related to kinases and compounds to improve the prediction accuracy of highly selective kinase inhibitors. Shen et al. [25] constructed a kinase-compound heterogeneous network using known activity data, which contains compound similarity information and kinase-compound activity information. Based on this heterogeneous network, a diffusion propagation method was proposed to predict the inhibition relationship of kinase compound activity. The experimental results show that the prediction accuracy of kinase compound activity can be improved by using the knowledge of kinase and compound domain to build an isomer network. Most related to our research work, Li et al. [20] recently presented a virtual kinase chemogenomic model for predicting the interaction profiles of kinase inhibitors against a panel of 391 kinases based on large-scale bioactivity data and the MTDNN algorithm. As a result of the high relatedness among kinases resulting from their promiscuousness and the transfer learning effect of MTDNN, the obtained model yields excellent pre-diction ability. The model consistently shows higher predictive performance than conventional single-task models, especially for kinases with insufficient activity data.

Despite the effectiveness of the existing methods for kinase inhibitors prediction, data scarcity issue remains an important challenge to the prediction performance of kinase inhibitors activity. However, most existing research works have ignored this issue, except [20] tries to alleviate the data scarcity problem by exploiting multi-task learning. It is worth noting that for most kinases, the known active and inactive compounds are often limited. Based on the Kinase SARfari database, and the Kinome data set published by Metz et al. [26], we collected and curated the data set consisting of 389 kinases, 32808 compounds, and 177676 biological activity data. We found from the datasets that a large number of kinases (77%) have a small number of samples with the range of 1–99. The limited training samples easily lead to overfitting of the prediction model, which greatly restricts the training quality and prediction performance of the model, and brings great challenges to the quality of virtual screening of kinase inhibitors based on machine learning. In addition, the multi-task learning model [20] exploited the relatedness among different kinase prediction tasks to improve the prediction performance of the model. However, the experimental results show that the prediction accuracy of a large number of small samples of kinases still needs to be improved as the literature [20] reported that the prediction performance of the multi-task deep learning method on validation data sets decreased significantly with the decrease of the sample data volume of the kinase pre-diction task.

To tackle the aforementioned data scarcity challenges of current approaches for kinase inhibitor activity prediction, in this study, we present a novel Meta-learning Inductive Logistic Matrix Completion (MetaILMC) to alleviate the data sparsity problem faced by PKI. Meta-learning [27] is a new learning paradigm for few-shot application scenarios that focuses on deriving prior knowledge across different learning tasks, to rapidly adapt to a new learning task with the prior and a small amount of training data. Recently, some research has been devoted to exploring meta-learning methods to solve the few-shot learning issues in biology or medicine, such as [35, 36]. To some extent, PKI with few shot samples can be formulated as a meta-learning problem. Specifically, each task is constructed for a kinase. From the tasks for kinases with sufficient training samples, the meta-learner learns a prior with strong generalization capacity during meta-training, such that it can be easily and quickly adapted to the new tasks of the kinase with scarce data during meta-testing. As MetaILMC allows the effective transfer of the prior knowledge learned from kinases with sufficient samples to kinases with a small number of samples, the proposed model can produce accurate predictions for kinases with limited data.

We compared the proposed method with other baselines on our collected and curated datasets. Experimental results show that MetaILMC has excellent performance for prediction tasks of kinases with few-shot samples and is significantly superior to the state-of-the-art method in terms of AUC, AUPR, etc., various performance metrics. Case studies also provided for two drugs to predict Kinase scores, further validating the proposed method's effectiveness and feasibility.

## Methods

### Data collection

Two open-accessed Kinase datasets are used to construct our experimental datasets. (1) The SARfari data set (http://wwwdev.ebi.ac.uk/chembl/sarfari/kinasesarfari) is an integrated chemogenomic workbench focused on kinases, which is composed of 54,189 compounds, 989 different kinase domains, and 532,155 Kinase-compound data points in the form of IC50, Ki, Kd, and other values. (2) The second data set, the Metz data set [26], contains 1498 compounds with known structures, 173 human kinases, and 107,791 pKi data points. The inhibition activity in the merged data set was converted to two classes: active (pKi /pKd/pIC50 ≥ 6) and inactive (pKi /pKd/pIC50 < 6). After the deletion of mutant kinases and kinases without both active and inactive data points, the final data set (named KinaseDB) contains over 182,447 data points between 388 kinases and 34,682 compounds.

**Fig. 1:**
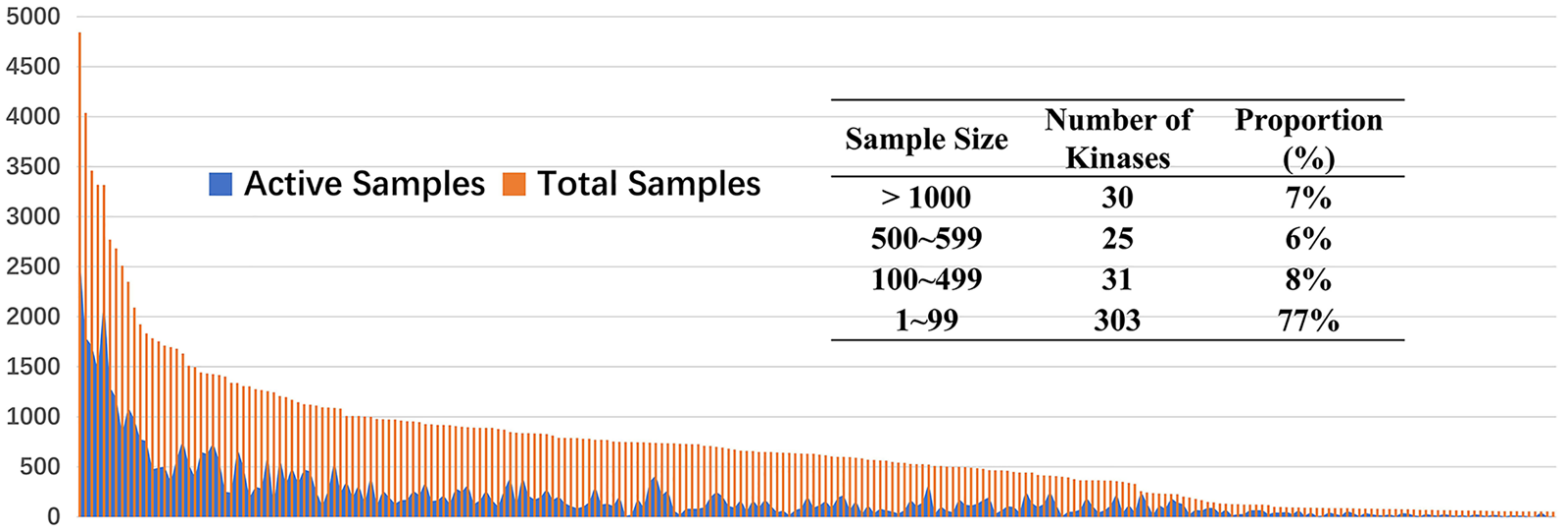
The samples statistics for 388 protein kinases in KinaseDB. The figure shows that the compounds statistics follow an obvious long tail distribution, i.e., only few kinases have many samples, majority of kinases just have a small number of samples. It is worth noting that a large number of kinases (303, 77%) have a small number of samples with the range of 1–99. Detailed information about the protein kinases and sample statistics can be found in Additional file 1: Table S.1

Figure 1 shows the statistics about the number of sample points contained for each kinase in our collected and curated datasets KinaseDB. It is easy to see that the statistics follow an obvious long-tail distribution, i.e., only a few kinases have many points, majority of kinases just have a small number of points. More specifically there are 30 kinases with more than 1000 samples, accounting for 7% of the total number of kinases, 25 kinases with 500 ~ 999 samples, accounting for 6% of the total number of kinases, 31 kinases with 100 ~ 499 samples, accounting for 8% of the total number of kinases, majority of 303 kinases with less than 100 samples, accounting for 77% of the total number of kinases.

### Problem formulation

This paper aims to tackle the issue of predicting the interaction profiles of kinase inhibitors against Kinome (hereinafter abbreviated as PKI). Considering with $P$ of $m$ kinases, $C$ of $n$ compounds, and $n\times m$ experimentally verified compound-kinase interaction matrix $\mathbf{T}\in {\left\{\mathrm{1,0},{\text{null}}\right\}}^{n\times m}$.$\mathbf{T}\left(i,j\right)=1$ if a compound $i$ is inhibitory active for a protein kinase $j$. $\mathbf{T}\left(i,j\right)=0$ if a compound $i$ is not inhibitory active for a protein kinase $j$. $\mathbf{T}\left(i,j\right)={\text{null}}$ if a compound $i$ is unknown inhibitory active for a protein kinase $j$. Let ${\Omega }^{+}=\left\{\left({c}_{i},{p}_{j}\right)|\mathbf{T}\left(i,j\right)=1, {c}_{i}\in C,{p}_{j}\in P\right\}$ be the set of inhibitory active pair. Similarly, we also have ${\Omega }^{-}=\left\{\left({c}_{i},{p}_{j}\right)|\mathbf{T}\left(i,j\right)=0, {c}_{i}\in C,{p}_{j}\in P\right\}$. Thus, PKI aims to establish a machine-learning-based model to predict the interaction profiles of any compound against Kinome using ${\Omega }_{{\text{tr}}}={\Omega }_{{\text{tr}}}^{+}\cup {\Omega }_{{\text{tr}}}^{-}$ (${\Omega }_{{\text{tr}}}^{+}\subseteq {\Omega }^{+}$, ${\Omega }_{{\text{tr}}}^{-}\subseteq {\Omega }^{-}$) as training data.

### Inductive logistic matrix completion for PKI

Generally, PKI can be modeled as a matrix completion (MC) for the partially observed matrix T. However, MC can only provide a solution of transductive learning, since the learned embeddings cannot generalize to unseen compounds, i.e., can only be used to predict T-related compound-kinase prediction problems. In the real application environment, PKI is required to have the ability of virtual screening, that is, given a new compound, predict the activity of the compound to Kinome. Therefore, an inductive learning model is desired to be established for PKI.

In this paper, inspired by the Inductive Matrix Completion (IMC) [28], we propose an Inductive Logistic Matrix Completion (ILMC) based model for PKI. Let $\mathbf{T}\in {\left\{\mathrm{1,0},{\text{null}}\right\}}^{n\times m}$ be the partial observed interaction matrix with $m$ kinases, $n$ compounds. ${\mathbf{X}}_{p}\in {\mathbb{R}}^{m\times {d}_{p}}$ and ${\mathbf{X}}_{c}\in {\mathbb{R}}^{n\times {d}_{c}}$ are the kinases and compounds feature matrices respectively (Later, in experimental section we will introduce the details of obtaining the feature matrices). ${\mathbf{X}}_{c}^{\mathrm{\top }}\left(i\right)\in {\mathbb{R}}^{{d}_{c}}$ and ${\mathbf{X}}_{p}^{\mathrm{\top }}\left(j\right)\in {\mathbb{R}}^{{d}_{p}}$ are the *i*-th compound and *j*-th kinase feature vector respectively. Then, the likelihood for PKI is defined as

1 $${\mathcal{L}}_{{\text{MLE}}}\left(\mathbf{T}|\mathbf{U},\mathbf{V}\right)={\prod }_{\left(i,j\right)\in {\Omega }_{{\text{tr}}}^{+}\cap {\Omega }_{{\text{tr}}}^{-}}{P}_{ij}^{{\mathbf{T}}_{i,j}}{\left(1-{P}_{ij}\right)}^{\left(1-{\mathbf{T}}_{i,j}\right)}$$

where the active probability ${P}_{ij}$ for the pair of compounds $i$ and protein kinase $j$ is defined as

2 $${P}_{ij}=\sigma \left({\mathbf{X}}_{c}\left(i\right),{\mathbf{X}}_{p}^{\top }\left(j\right)|\mathbf{U},\mathbf{V}\right)=\frac{{\text{exp}}\left({\text{NN}}\left({\mathbf{X}}_{c}\left(i\right)|\mathbf{U}\right){\text{NN}}\left({\mathbf{X}}_{p}^{\top }\left(j\right)|\mathbf{V}\right)\right)}{1+{\text{exp}}\left({\text{NN}}\left({\mathbf{X}}_{c}\left(i\right)|\mathbf{U}\right){\text{NN}}\left({\mathbf{X}}_{p}^{\top }\left(j\right)|\mathbf{V}\right)\right)}$$

and $\mathbf{U},\mathbf{V}$ are the learnable parameters of MLPs. Thus, PKI is formulated as a maximum likelihood estimation (MLE) problem as follows.

3 $${{\text{max}}}_{\mathbf{U},\mathbf{V}}{\text{ln}}{\mathcal{L}}_{{\text{MLE}}}\left(\mathbf{T}|\mathbf{U},\mathbf{V}\right)={\sum }_{\left(i,j\right)\in {\Omega }_{{\text{tr}}}^{+}\cap {\Omega }_{{\text{tr}}}^{-}}\left({\mathbf{T}}_{i,j}{\text{ln}}{P}_{ij}+\left(1-{\mathbf{T}}_{i,j}\right){\text{ln}}\left(1-{P}_{ij}\right)\right)+\lambda \left({\Vert \mathbf{U}\Vert }_{F}^{2}+{\Vert \mathbf{V}\Vert }_{F}^{2}\right)$$

It is worth pointing out that since the learned feature transformation MLPs i.e., ${\text{NN}}\left(\bullet |\mathbf{U}\right)$ and ${\text{NN}}\left(\bullet |\mathbf{V}\right)$ can generalize to unseen kinase and compound, ILMC is an inductive learning model.

### Meta inductive logistic matrix completion for few shots PKI

According to the statistical results of the kinase dataset (see Fig. 1), a majority of kinases have only a few samples. Obviously, due to the lack of sufficient samples for model training, the prediction performance of these few-shot kinase tasks will be degraded. The data sparsity thus raises a challenge for the prediction of kinase inhibitors against Kinome using ILMC.

To alleviate the data scarcity problem, in this paper, we propose a novel meta-learning approach, named MetaILMC, for the prediction of the interaction profiles of kinase inhibitors against Kinome. MetaILMC is a gradient optimization-based meta-learning method that leverages the idea of MAML [27] to establish its basic architecture. The basic idea underlying MetaILMC is to train the model’s initial parameters with sufficient sample tasks (we call them head tasks) such that the model has maximal performance on a new task after the parameters have been adapted through one or more gradient steps computed with a small number of samples from that new task.

**Fig. 2:**
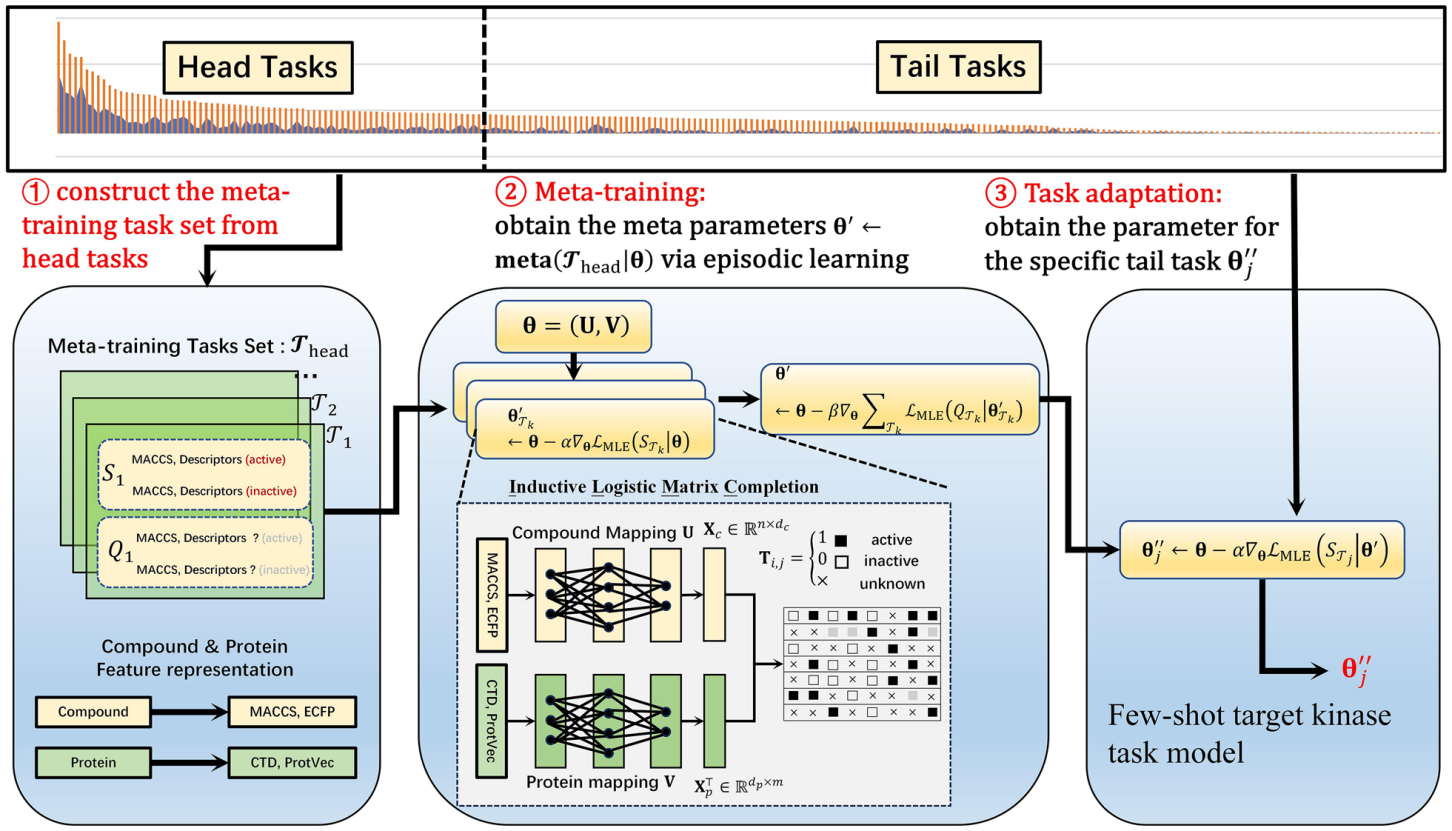
The overall framework of MetaILMC. MetaILMC consists of two phases: meta-training and meta-test (few-shot samples adaptation). In the meta-training phase, multiple kinases with sufficient samples are adopted as meta-training tasks to obtain a well-initialized model which could be fast adapted to a new kinase with limited data. In the adaptation phase, a few (e.g., less than 5) known active and inactive samples from a new target kinase are used to fine-tune the model on this kinase to capture its specific model

Generally, MetaILMC consists of two phases: meta-training and meta-test (few-shot samples adaptation). In the meta-training phase, multiple kinases with sufficient samples are adopted as meta-training tasks to obtain a well-initialized model that could be fast adapted to a new kinase with limited data. In the adaptation phase, a few (e.g., less than 5) known active and inactive samples from a new target kinase are used to fine-tune the model on this kinase to capture its specific model. With the transferability and fast adaptability between meta-training tasks and the new tasks with few-shot samples, MetaILMC can be applied to mitigate the data scarcity issue. The following Fig. 2 gives the overall framework of MetaILMC.

Before formally describe and define MetaILMC, we introduce some notations. In our MetaILMC framework, each task ${\mathcal{T}}_{k}$ is constructed for a kinase$k$. Let $\mathcal{T}={\mathcal{T}}_{{\text{head}}}\cup {\mathcal{T}}_{{\text{tail}}}$ (${\mathcal{T}}_{{\text{head}}}\cap {\mathcal{T}}_{{\text{tail}}}=\varnothing$) be the total tasks set. ${\mathcal{T}}_{{\text{head}}}=\left\{{\mathcal{T}}_{1},{\mathcal{T}}_{2},\dots ,{\mathcal{T}}_{{\ell}}\right\}$ denotes the set of tasks with sufficient samples. ${\mathcal{T}}_{{\text{tail}}}=\left\{{\mathcal{T}}_{{\ell}+1},{\mathcal{T}}_{{\ell}+2},\dots ,{\mathcal{T}}_{m}\right\}$ denotes the set of tasks with few-shot samples. As defined in section Problem Formulation, ${\Omega }^{+}$ (${\Omega }^{-}$) is the set of inhibitory active (inactive) pair. Each task ${\mathcal{T}}_{k}=\left\{{S}_{{\mathcal{T}}_{k}},{Q}_{{\mathcal{T}}_{k}}\right\}$ for a kinase *k* consists of a support compound set ${S}_{{\mathcal{T}}_{k}}$ and a query compound set ${Q}_{{\mathcal{T}}_{k}}$ where${S}_{{\mathcal{T}}_{k}}\subset {\Omega }_{{\mathcal{T}}_{k}}^{+}\cup {\Omega }_{{\mathcal{T}}_{k}}^{-}$, ${Q}_{{\mathcal{T}}_{k}}\subset {\Omega }_{{\mathcal{T}}_{k}}^{+}\cup {\Omega }_{{\mathcal{T}}_{k}}^{-}$ sampled from the set of active or inactive compounds for the kinase *k*, such that the support and query compounds are mutually exclusive, i.e.,${S}_{{\mathcal{T}}_{k}}\cap {Q}_{{\mathcal{T}}_{k}}=\varnothing$.

Specifically, the MetaILMC consists of two following phases.

- (1). Meta-training Phase (${\mathbf{\theta }}^{{\text{'}}} \leftarrow \mathbf{m}\mathbf{e}\mathbf{t}\mathbf{a}\left({\mathcal{T}}_{{\text{head}}}|{\varvec{\uptheta}}\right)$)

Starting with random initializing parameters ${\varvec{\uptheta}}$, the meta-training algorithm ${\mathbf{\theta }}^{{\text{'}}}$ yields the learned meta parameters ${\mathbf{\theta }}^{{\text{'}}}$ using head tasks ${\mathcal{T}}_{{\text{head}}}$ as training tasks. The parameters ${{\varvec{\uptheta}}}{\prime}$ learned by the ${\text{meta}}\left(\bullet \right)$ algorithm contain the prior knowledge of all head tasks which is desired to be generalized to all tail tasks. Specifically, let ${D}_{{\mathcal{T}}_{k}}$ be the set of compound-kinase pair related to the task ${\mathcal{T}}_{k}$. ${\varvec{\uptheta}}=\left(\mathbf{U},\mathbf{V}\right)$ are the parameters for ILMC. The data likelihood of ILMC for ${D}_{{\mathcal{T}}_{k}}$ under ${\varvec{\uptheta}}$ is defined as

4 $${\mathcal{L}}_{{\text{MLE}}}\left({D}_{{\mathcal{T}}_{k}}|{\varvec{\uptheta}}\right)=p\left({D}_{{\mathcal{T}}_{k}}|\mathbf{U},\mathbf{V}\right)={\sum }_{\left({c}_{i},{p}_{k}\right)\in {D}_{{\mathcal{T}}_{k}}}\left({\mathbf{T}}_{i,k}{\text{ln}}{P}_{ik}+\left(1-{\mathbf{T}}_{i,k}\right){\text{ln}}\left(1-{P}_{ik}\right)\right)+\lambda \left({\Vert \mathbf{U}\Vert }_{F}^{2}+{\Vert \mathbf{V}\Vert }_{F}^{2}\right)$$

For each head task ${\mathcal{T}}_{k}=\left\{{S}_{{\mathcal{T}}_{k}},{Q}_{{\mathcal{T}}_{k}}\right\}\in {\mathcal{T}}_{{\text{head}}}$. The meta-learner adapts the global prior ${\varvec{\uptheta}}$ to task-specific parameters ${{\varvec{\uptheta}}}_{{\mathcal{T}}_{k}}{\prime}$ w.r.t. the loss on the support set ${S}_{{\mathcal{T}}_{k}}$.

5 $${\mathbf{\theta }}^{{\text{'}}} \leftarrow {\varvec{\uptheta}}-\alpha {\nabla }_{{\varvec{\uptheta}}}{\mathcal{L}}_{{\text{MLE}}}\left({S}_{{\mathcal{T}}_{k}}|{\varvec{\uptheta}}\right)$$

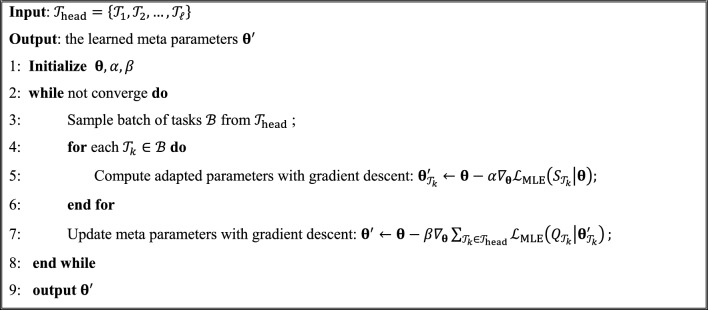


Equation (5) is called the inner-loop update process of meta-training. The updated ILMC parameters after several steps on data from the support set ${S}_{{\mathcal{T}}_{k}}$ can be expressed where α is the inner-loop learning rate. The $\alpha$ is fixed as a hyperparameter and shared by all meta-training tasks. We will investigate the effect of $\alpha$ on model performance in the experimental section. For simplicity of notation, one gradient update is shown in Eq (5), but using multiple gradient updates is allowed as well.

For each query set ${Q}_{{\mathcal{T}}_{k}}$, the loss under the task-specific parameters ${{\varvec{\uptheta}}}_{{\mathcal{T}}_{k}}{\prime}$ is calculated and the backward propagation is exploited to update the global ${\varvec{\uptheta}}$ using the loss sum of all meta-training tasks.

6 $${{\varvec{\uptheta}}}^{\boldsymbol{^{\prime}}}\leftarrow {\varvec{\uptheta}}-\beta {\nabla }_{{\varvec{\uptheta}}}{\sum }_{{\mathcal{T}}_{k}\in {\mathcal{T}}_{{\text{head}}}}{\mathcal{L}}_{{\text{MLE}}}\left({Q}_{{\mathcal{T}}_{k}}|{\varvec{\uptheta}}-\alpha {\nabla }_{{\varvec{\uptheta}}}{\mathcal{L}}_{{\text{MLE}}}\left({S}_{{\mathcal{T}}_{k}}|{\varvec{\uptheta}}\right)\right)$$

Equation (6) is called the outer-loop update process of meta-training where $\beta$ is called the outer-loop learning rate which is fixed as a hyperparameter. We will investigate the effect of $\beta$ on model performance in the experimental section. The following Algorithm 1 describes the complete procedure of meta-training.

- **(2). Few-shot Adaptation Phase** (${{\varvec{\uptheta}}}_{j}^{{\prime}{\prime}}\leftarrow \mathbf{a}\mathbf{p}\mathbf{t}\left({\mathcal{T}}_{j}|{S}_{{\mathcal{T}}_{j}},{{\varvec{\uptheta}}}{\prime}\right)$)

For each tail task ${\mathcal{T}}_{j}\in {\mathcal{T}}_{{\text{tail}}}$, the support set ${S}_{{\mathcal{T}}_{j}}$ still contains a small number of active and inactive compounds for the kinase *j*. The MetaILMC adapts the prior ${{\varvec{\uptheta}}}{\prime}$ learned during meta-training stage via one or a few gradient steps w.r.t. its support set ${S}_{{\mathcal{T}}_{j}}$ and finally yields the parameters ${{\varvec{\uptheta}}}_{j}^{{\prime}{\prime}}$ specific to the task ${\mathcal{T}}_{j}$.

7 $${{\varvec{\uptheta}}}_{j}^{{\prime}{\prime}}\leftarrow {\varvec{\uptheta}}-\alpha {\nabla }_{{\varvec{\uptheta}}}{\mathcal{L}}_{{\text{MLE}}}\left({S}_{{\mathcal{T}}_{j}}|{{\varvec{\uptheta}}}^{\boldsymbol{{\prime}}}\right)$$

Now, each few-shot kinase prediction task ${\mathcal{T}}_{j}$ has the model parameters ${{\varvec{\uptheta}}}_{j}^{{\prime}{\prime}}=\left({\mathbf{U}}_{j}^{{\prime}{\prime}},{\mathbf{V}}_{j}^{{\prime}{\prime}}\right)$. When a new compound ${\mathbf{x}}_{{\text{new}}}$ is input, active probability ${\mathbf{x}}_{{\text{new}}}$ for kinase *j* can be predicted by:

8 $${P}_{{\text{new}},j}=\sigma \left({\mathbf{x}}_{{\text{new}}},{\mathbf{X}}_{p}^{\top }\left(j\right)|{\mathbf{U}}_{j}^{{\prime}{\prime}},{\mathbf{V}}_{j}^{{\prime}{\prime}}\right)=\frac{{\text{exp}}\left({\mathbf{x}}_{{\text{new}}}{\mathbf{U}}_{j}^{{\prime}{\prime}}{{\mathbf{V}}_{j}^{{\prime}{\prime}}}^{\top }{\mathbf{X}}_{p}^{\top }\left(j\right)\right)}{1+{\text{exp}}\left({\mathbf{x}}_{{\text{new}}}{\mathbf{U}}_{j}^{{\prime}{\prime}}{{\mathbf{V}}_{j}^{{\prime}{\prime}}}^{\top }{\mathbf{X}}_{p}^{\top }\left(j\right)\right)}$$

## Results and discussion

### Experimental setup

As described in Sect. "Methods", we collected and preprocessed the experimental dataset based on the SARfari and Metz [26] data sets. The preprocessed data set is denoted as KinaseDB which finally contains over 182,447 bioactivity data points between 388 kinases and 34,682 compounds (see Additional file 1: Table S.1 for the detailed information and statistics of KinaseDB).

In addition, to further highlight the long-tail nature of the dataset, we establish a long-tail dataset based on KinaseDB. Specifically, we choose 27 kinases with sufficient samples as head kinases. Each head kinase has 500 active points and 500 inactive points as training samples. Then, the other 265 kinases are considered as tail kinases each of which has few-shot samples. Each tail kinase has 5 active points and 5 inactive points as training samples. For tail kinases, all compounds except those selected as active and inactive points are considered test samples. The preprocessed long-tail dataset is referred to as LTKinaseDB.

The chemical structure (SMILES format) of a compound contains a large amount of physicochemical property information. Therefore, for the structural features of the compounds, we assembled the chemical structure information (SMILES format) from the merged dataset. We use RDKit (http://www.rdkit.org/) to compute the MACCS fingerprints for all of the compounds, and each compound’s length is 167 bits. We use the Conjoint Triad Descriptors (CTD) method [31] to compute the distribution of amino acid properties in the protein sequences, the 20 amino acids were clustered into seven classes according to their dipoles and volumes of the side chains. The conjoint triad descriptors consider the property of amino acid along with its adjacent amino acids as one single unit of three amino acids, thus the dimension of one protein should be 7*7*7, you can use CTD in pfeature website (https://webs.iiitd.edu.in).

The experimental code is implemented based on the open-source machine learning framework Pytorch (https://pytorch.org). All experiments are carried out on Windows 10 operating system with a Dell Precision T5820 workstation computer of an intel W-2145 8 cores, 3.7 GHz CPU, and 64G memory. All datasets and experimental code are available from https://github.com/ljatynu/MetaILMC/.

### Baselines

In the experiments, our proposed methods are compared with the other five baselines which included two deep-learning based baselines, MTDNN [20], MolTrans [29] and three traditional machine learning baselines, support vector machine (SVM), random forest (RF), and k-nearest neighbors (KNN) [33]. Particularly, MolTrans [29] exploited a sub-structural pattern mining algorithm and interaction modeling module for more accurate and interpretable DTI prediction. MTDNN [20] is a multitask deep neural network-based model for PKI. Li et al. [20] have showed that MTDNN consistently shows higher predictive performance than conventional single-task models, especially for kinases with insufficient activity data in the prediction of highly potent inhibitors of 391 human kinases by exploiting high relatedness among various kinases predictive tasks.

### Predictive performance of ILMC

We first verify the global predictive performance of ILMC on KinaseDB. The global means that we are not evaluating the predictive performance of ILMC for a single kinase. The 10-Fold- Cross Validation (10-FCV) is used to evaluate the performance of ILMC on KinaseDB. In 10-FCV, the known compound-kinase pairs (active or inactive) are randomly divided into 10 different subsets. A part of them is considered the testing set and the rest 9 divisions are considered the training set. The area under the receiver operating characteristic curve (AUC) and the area under the precision-recall curve (AUPR) are used to evaluate the performance of ILMC. To evaluate the performance of ILMC more comprehensively, we also use BA (balanced accuracy), Precision, Recall and the F1-score to verify the performance of the model. The final results are the average results over 10 experiments. ILMC adopts 3-layer MLPs (167-128-64 and 343-128-64) to make feature transformations for kinase and compounds respectively. To explore the effectiveness of other feature representation methods for compounds and proteins in terms of model generalization ability. In the experiment, we also validated the predictive performance of ILMC when using extended connectivity fingerprinting (ECFP) for drugs and the ProtVec for proteins. ECFP used the settings of radius = 2 and nBits = 256 to obtain compound features. For ProtVec, we obtain the pre-trained protein features from biovec (https://github.com/kyu999/biovec).

Table 1 shows the comparison results under various evaluation criteria. Generally, the predictive performance of deep learning methods is superior to traditional machine learning baselines. MTDNN achieves the best performance. Two ILMCs, ILMC(ECFP + ProtVec) and ILMC(MACCS + CTD) achieve desirable performance as well which is slightly lower than that of MTDNN and MolTrans. At the same time, we also note that two ILMC models using two different feature representations, i.e., ILMC(ECFP + ProtVec) and ILMC(MACCS + CTD) achieved comparable prediction results.

**Table 1:** Performance comparison of different methods on KinaseDB (10-FCV, global evaluation model)

	AUC	AUPR	BA	RECALL	PRECISION	F1
SVM	0.6098	0.6655	0.6098	0.2397	0.6098	0.3738
KNN	0.817	0.7951	0.817	0.7388	0.817	0.7531
RF	0.8088	0.7989	0.8088	0.6975	0.8088	0.7469
MolTrans [29]	0.9297	0.8718	**0.8603**	0.7751	0.7267	**0.8013**
MTDNN [20]	**0.9302**	**0.8735**	0.8424	0.7708	0.8080	0.7889
ILMC(MACCS + CTD)	0.9290	0.8496	0.8304	**0.7800**	**0.8090**	0.7695
ILMC(ECFP + ProtVec)	0.9270	0.8595	0.8439	0.7795	0.8046	0.7891

The best results are shown in bold, the rank 2 score is marked by underline

### Data scarcity degenerates the performance of both ILMC and MTDNN

To simulate the circumstances of few-shot learning, each tail task in LTKinaseDB has only 5 active points and 5 inactive points. Each head task instead has 500 active points and 500 inactive points. Four experimental methods were trained on LTKinaseDB, all compounds except LTKinaseDB are considered as test samples. Table 2 shows the performance of ILMCs, MolTrans, and MTDNN on tail tasks decreased significantly compared with the results on head tasks. Few-shot samples degenerate significantly the performance of these models.

**Table 2:** Performance comparison of ILMC & MTDNN on head & tail kinase of LTKinaseDB (global evaluation model)

		AUC	AUPR	BA	RECALL	PRECISION	F1
ILMC (MACCS + CTD)	Head	0.8386	0.7919	0.7571	0.7590	0.7062	0.7317
	Tail	0.7204	0.4509	0.6621	0.6423	0.3995	0.4926
ILMC (ECFP + ProtVec)	Head	0.8549	0.8193	0.7876	0.7832	0.7448	0.7635
	Tail	0.7049	0.4646	0.6595	0.6675	0.4102	0.5277
MTDNN [20]	Head	0.8753	0.8401	0.7945	0.7868	0.7552	0.7706
	Tail	0.7034	0.4448	0.6477	0.6639	0.3724	0.4772
MolTrans [29]	Head	0.8453	0.8041	0.7643	0.7679	0.6774	0.7488
	Tail	0.7253	0.4442	0.6805	0.6908	0.3620	0.5377

Based on the experimental results, we infer that the MTDNN, MolTrans, and ILMCs achieve high global accuracy for the task of kinase activity prediction. However, we also found that there was a significant difference in the predictive performance of these models on head and tail tasks. The issue of few-shot sample learning brings great challenges to the predictive performance of kinase inhibitors against Kinome.

### Effect of parameter setting on MetaILMC prediction performance

The number of meta-training tasks, the inner-loop learning rate $\alpha$, the gradient descent steps of inner-loops, and the outer-loop learning rate $\beta$ all affect the training results of the meta parameters. In this section, we conducted experiments to investigate the effect of parameter setting on MetaILMC prediction performance.

Table 3 results show the effect of the number of meta-training learning tasks on the performance of MetaILMC. From the results, we can see that with the increase in the number of tasks involved in meta-training, the prediction performance of the model on target tasks with few-shot samples is also continuously improved. This result is consistent with the intuition that meta-learning can effectively achieve knowledge transfer across tasks.

**Table 3:** The effect of the number of meta-training tasks on performance of MetaILMC

	The number of meta-training tasks		
	5 tasks	15 tasks	27 tasks
AUC	0.7937	0.8459	0.8754
AUPR	0.5974	0.6761	0.7265

The inner-loop learning rate $\alpha$, the gradient descent steps of inner-loops, and the outer-loop learning rate $\beta$ all affect both the generalization and convergence speed of MetaILMC (as the effect of the gradient descent steps of outer-loops has no regular experimental results for the prediction performance, we omit the results here). Tables 4, 5, 6 sshowsthe experimental results of the effect of various parameter settings on MetaILMC prediction performance. According to the experimental results, in the following experiments, MetaILMC adopts $\alpha =0.01$, $\beta =0.01,$, and 4 as the gradient descent steps of inner-loops to carry on experiments.

**Table 4:** The effect of various inner-loop learning rate $\boldsymbol{\alpha }$ on performance of MetaILMC

	Inner-loop learning rate $\alpha$				
	0.1	0.05	0.01	0.001	0.0001
AUC	0.8518	0.859	0.8754	0.8365	0.8304
AUPR	0.6945	0.702	0.7265	0.6599	0.6465

**Table 5:** The effect of various outer-loop learning rate ${\varvec{\beta}}$ on performance of MetaILMC

	Outer-loop learning rate $\beta$				
	0.1	0.01	0.005	0.001	0.0001
AUC	0.8446	0.8754	0.8538	0.8515	0.7737
AUPR	0.6885	0.7265	0.7017	0.7005	0.5322

**Table 6:** The effect of the gradient descent steps of inner-loops on performance of MetaILMC

	# Gradient descent steps				
	1	2	3	4	5
AUC	0.8531	0.8552	0.8615	0.8754	0.8577
AUPR	0.6863	0.6932	0.7005	0.7265	0.6988

### MetaILMC can improve the performance in few-shot learning circumstances

Given the difference in prediction between head and tail kinases mentioned above, we proposed MetaILMC to improve the prediction performance for tail kinases. In the *meta-training* phase, 27 head kinases with sufficient samples in LTKinaseDB were used as the meta-training tasks to train MetaILMC. Specifically, in each epoch of meta training, each head task ${\mathcal{T}}_{k}$ was adapted by feeding with randomly selected 5 active points and 5 inactive points as ${S}_{{\mathcal{T}}_{k}}$ set, and 10 active points and 10 inactive points as ${Q}_{{\mathcal{T}}_{k}}$ set. In the *meta-testing* phase, 265 tail kinases with few-shot samples in LTKinaseDB were used as the meta-testing tasks to evaluate the predictive performance of MetaILMC. Specifically, the few-shot support set (5 active points and 5 inactive points) of each tail task was utilized to adapt parameters ${\varvec{\uptheta}}$ of MetaILMC via a small number of gradient descent steps using Eq. (1), then all remaining samples of the tail task were used as test set to evaluate the predictive performance of the adapted MetaILMC. Since under the framework of meta-learning, each tail task has its predictive model, a local evaluation model is adopted to evaluate the performance of various methods, i.e., the performance of each task is evaluated by the test set belonging to the corresponding kinase. The final performance of MetaILMC was evaluated by the average performance of 265 tail tasks.

We compared the MetaILMCs, i.e., MetaILMC(MACCS + CTD) and MetaILMC(ECFP + ProtVec), to the other baselines (including ILMC). All compared baselines used LTKinaseDB as training data to train the models and average the predictive results of tail kinases to obtain the final performance. To verify the generalization ability and transfer learning ability of MetaILMC, we compared it with other recent baselines, including MTDNN [20] (a multi-task learning model), MolTrans [29], and MetaMGNN [30] (a meta-learning model). MTDNN and MetaMGNN use the entire long tail dataset as a train set, consistent with ILMC, to predict and calculate AUC values on each tail kinase test set. For the single-task model, random forest, SVM, and KNN algorithms were selected, and only 5 active points and 5 inactive points of a single tail kinase were used as the train set each time. Then, the prediction performance is evaluated on the test set of each tail kinase. The comparison results are shown in Table 7. It should be mentioned that due to the superior performance of MetaILMC (MACCS + CTD) over MetaILMC (ECFP + ProtVec) under few-shot learning circumstances, in the following we only provide the experimental results of MetaILMC (MACCS + CTD) as the comparison experimental results of the MetaILMC method. The detailed compare results of various methods on each tail task of LTKinaseDB can be found in the Additional file 2: Table S.2-AUC, Additional file 3: Table S.3-AUPR, Additional file 4: Table S.4-PRECISION, Additional file 5: Table S.5-RECALL, Additional file 6: Table S.6-BA, Additional file 7: Table S.7-F1.

**Table 7:** Performance comparison of various methods on tail kinase of LTKinaseDB (local evaluation model)

	AUC	AUPR	BA	RECALL	PRECISION	F1
MetaILMC (MACCS + CTD)	**0.8754**	**0.7265**	**0.8215**	**0.9170**	**0.6432**	**0.6636**
MetaILMC (ECFP + ProtVec)	0.8461	0.6726	0.7972	0.8896	0.5429	0.6018
ILMC	0.7724	0.5401	0.7319	0.8196	0.4726	0.5202
MTDNN [20]	0.7403	0.5044	0.6971	0.8605	0.4383	0.4906
SVM	0.5541	0.5153	0.5541	0.4599	0.3925	0.3016
KNN	0.5586	0.4958	0.5586	0.6097	0.2916	0.3554
RF	0.5976	0.5139	0.5976	0.6090	0.3263	0.3870
MolTrans [29]	0.6366	0.6245	0.6124	0.8042	0.4862	0.4220
MetaMGNN[30]	0.7280	0.6294	0.7280	0.8037	0.4334	0.5099

The best results are shown in bold, the second score is marked by underline

**Fig. 3:**
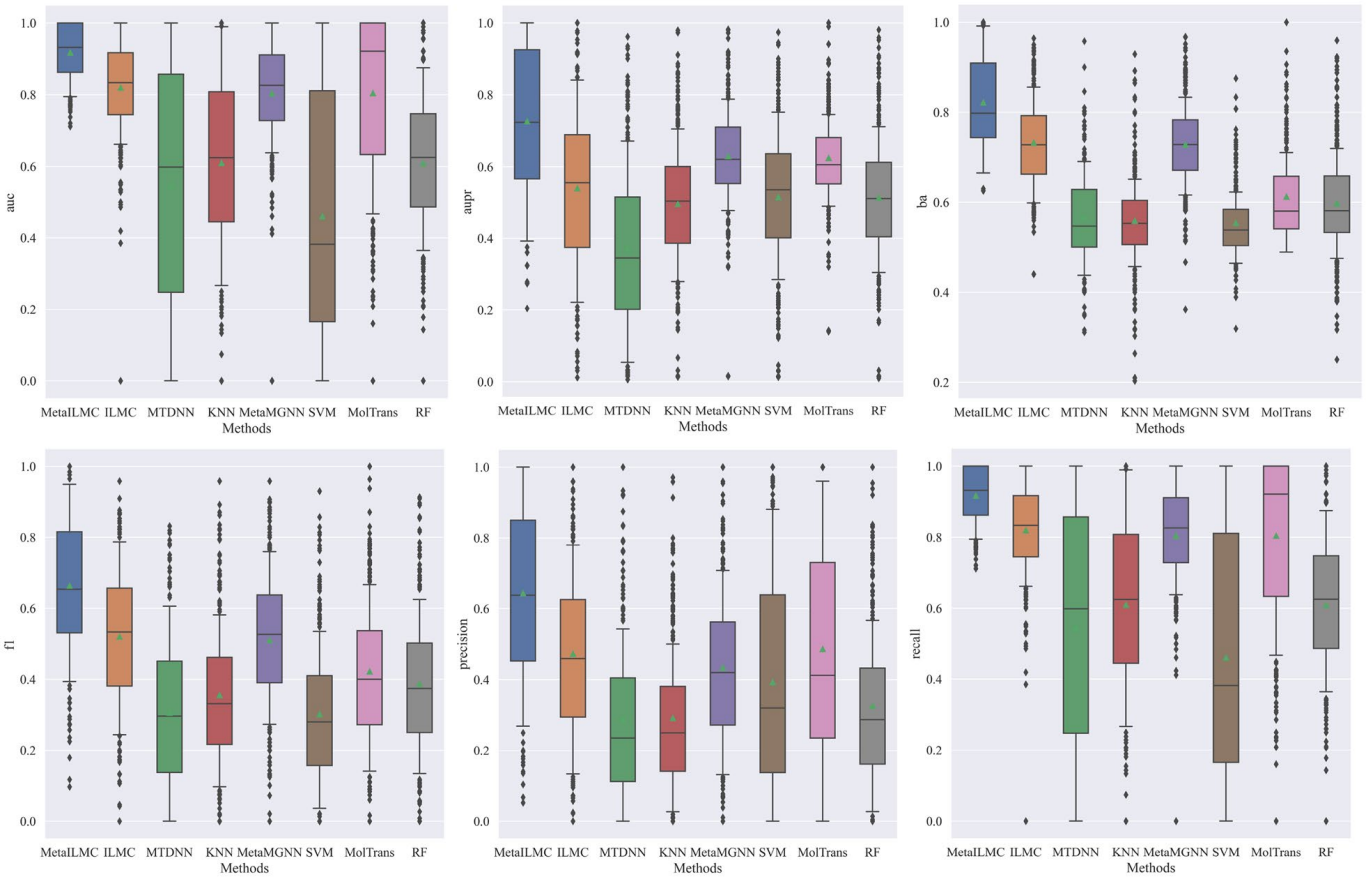
Performance comparison between Meta-ILMC and other comparison methods. Figures a ~ f respectively represents the performance of different algorithms under AUC, AUPR, BA, F1, RECALL, PRECISION performance metrics. Black lines in boxes depict the median, triangle depict the average, and boxes illustrate the interquartile range (IQR) of the distribution. Whiskers extent to 1.5·IQR from the median

We also present the box plots as shown in Fig. 3 to compare the performance of various on BA, AUC, F1, RECALL, PRECISION and AUPR. From Fig. 3, MetaILMC has the highest average and median among all methods in all performance indicators. In Fig. 3(b), the average AUC of MetaILMC is greater than 0.85 and higher than those of the comparison methods, in addition, the prediction results of MetaILMC for all tail kinases are clustered between 0.66 and 1, indicating the superior performance of the MetaILMC model in the prediction of kinase inhibitors with LTKinaseDB, when we just have a small number of training data points, this model also can achieve better prediction performance, we can get the same conclusion from another figure in Fig. 3. Take a look at the images in Fig. 3 as a whole, MetaILMC has the best prediction performance in all indicators, and prediction results are concentrated, moreover, it has fewer outliers, which indicates that MetaILMC has high robustness and can perform better for different kinases with small and different training points.

The above experimental results demonstrate that in few-shot learning circumstances, MetaILMC outperforms all baseline models under various evaluation metrics. Compared with other methods, MetaILMC has a good ability to learn task priori, and can effectively improve the prediction performance of kinases with few samples.

**Table 8:** Case Study

Drug	Kinase	Predicted Scores	pKd
Dasatinib	Q9Y4K4	0.997994542	(7,)
	Q8N4C8	0.99648869	(6,7]
	P07948	0.99596256	(7,)
	Q92918	0.992815614	(6,7]
	P54753	0.991856694	(7,)
	P36896	0.99170506	(6,7]
	P48730	0.990989685	/
	P54760	0.990192294	(7,)
	P51451	0.987114549	(7,)
	P43403	0.986486316	/
Sunitinib	O95819	0.99972111	(6,7]
	Q96SB4	0.999653816	(6,7]
	P23443	0.999647975	(7,)
	O15530	0.999610126	(5,6]
	O00444	0.999576867	(6,7]
	Q9H2X6	0.999403119	(7,)
	O94806	0.999173343	(6,7]
	P52333	0.998999894	(5,6]
	P22607	0.998657823	(6,7]
	O60285	0.998578429	(7,)

The top-10 potential kinases candidates detected by our models for Dasatinib and Sunitinib, pKd value from HMS LINCS dataset, if pKd > 6, kinase is active

### Case study

To further demonstrate the accuracy of our proposed model for predicting unobserved compounds, we chose two anticancer drugs approved by the US FDA, Dasatinib [32] and Sunitinib [33] as case studies. We used the ILMC model based on kinaseDB dataset to predict head kinases and the MetaILMC model based on the LTKinaseDB dataset to predict tail kinases, then prioritized all kinases using their predicted scores. We verified the top-10 human kinases’s predictions with HMS LINCS dataset [34]. As shown in Table 8, both eight kinases for Dasatinib and Sunitinib were supported by direct evidence. The results prove that our proposed model is effective.

## Conclusion

Protein kinases play critical roles in numerous human diseases. Therefore, developing new, efficient and safe small-molecule kinase inhibitors has become an important topic in the field of drug research and development. Machine learning-based methods have low experiment costs, high efficiency, and can effectively narrow the scope of experiments and reduce experimental blindness. However, the existing research works have neglected the issue of few-shot samples which is a common challenge for the majority of kinases. To tackle the issue of few-shot machine learning, meta-learning trains the meta-model over a large number of tasks with limited training samples in each task. The meta-model parameters are optimized via gradient descent according to the adaption performance on these tasks, so the learned model can be fast adapted and generalized well on new tasks with limited samples. Inspired by meta-learning, in this study, we develop a novel multi-task meta-learning MetaILMC to learn a well-generalized model that enables fast adaptation on new tasks with limited samples.

Experimental results show that MetaILMC has excellent performance for prediction tasks of kinases with few-shot samples and is significantly superior to the state-of-the-art method in terms of AUC, AUPR, etc., various performance metrics. Case studies also provided for two drugs to predict Kinase scores, further validating the effectiveness and feasibility of the proposed method. We believe that the proposed MetaILMC can be used to improve the performance of the prediction method of kinase inhibitor activity and actively promote the development of kinase inhibitors.

### Supplementary Information

**Additional file 1:Table S.1.** The detailed information and statistics of KinaseDB.

**Additional file 2: Table S.2.** The detailed compare results of various methods on each tail task of LTKinaseDB in terms of AUC.

**Additional file 3: Table S.3.** The detailed compare results of various methods on each tail task of LTKinaseDB in terms of AUPR.

**Additional file 4: Table S.4.** The detailed compare results of various methods on each tail task of LTKinaseDB in terms of PRECISION.

**Additional file 5: Table S.5.** The detailed compare results of various methods on each tail task of LTKinaseDB in terms of RECALL.

**Additional file 6: Table S.6.** The detailed compare results of various methods on each tail task of LTKinaseDB in terms of BA.

**Additional file 7: Table S.7.** The detailed compare results of various methods on each tail task of LTKinaseDB in terms of F1.

## Data Availability

The implemented code and experimental dataset are available online at https://github.com/ljatynu/MetaILMC/
