# Supplementary material for: Meta-learning-based Inductive logistic matrix completion for prediction of kinase inhibitors
Source: J Cheminform. 2024 Apr 16;16:44. doi: 10.1186/s13321-024-00838-9 (PMC11301988; doi:10.1186/s13321-024-00838-9)
Supplement: Supplementary file 1 — Additional file 1:Table S.1. The detailed information and statistics of KinaseDB. [file 13321_2024_838_MOESM1_ESM.docx]

**Table S.1**

**The detailed information of the protein kinases and sample statistics**

| **UniPort ID** | **Name** | **Family** | **Organism** | **Active/Inactive points** |
| --- | --- | --- | --- | --- |
| Q8IVH8 | hMAP4K3 | STE | Homo sapiens | 12/30 |
| Q16539 | hp38a | CMGC | Homo sapiens | 2309/864 |
| O15146 | hMUSK | TK | Homo sapiens | 11/33 |
| Q16816 | hPHKG1 | CAMK | Homo sapiens | 44/57 |
| Q15375 | hEPHA7 | TK | Homo sapiens | 7/37 |
| P21803 | mFGFR2 | TK | Mus musculus | 11/18 |
| P32298 | hGRK4 | AGC | Homo sapiens | 4/4 |
| P29317 | hEPHA2 | TK | Homo sapiens | 114/663 |
| Q8TDX7 | hNEK7 | Other | Homo sapiens | 0/42 |
| P48729 | hCSNK1A1 | CK1 | Homo sapiens | 136/863 |
| P41240 | hCSK | TK | Homo sapiens | 17/73 |
| P90584 | plPfmrk | CMGC | Plasmodium falciparum | 12/114 |
| P35916 | hVEGFR3 | TK | Homo sapiens | 304/648 |
| Q64725 | rSYK | TK | Rattus norvegicus | 101/429 |
| Q9NR20 | hDYRK4 | CMGC | Homo sapiens | 32/510 |
| Q9Y463 | hDYRK1B | CMGC | Homo sapiens | 128/309 |
| Q8IU85 | hCAMK1D | CAMK | Homo sapiens | 45/345 |
| P05131 | bPKAb | AGC | Bos taurus | 21/131 |
| Q9WUD9 | rSRC | TK | Rattus norvegicus | 29/15 |
| P11802 | hCDK4 | CMGC | Homo sapiens | 733/780 |
| P17948 | hVEGFR1 | TK | Homo sapiens | 408/367 |
| P35590 | hTIE1 | TK | Homo sapiens | 13/28 |
| P16591 | hFER | TK | Homo sapiens | 124/385 |
| Q6ZN16 | hMAP3K15 | STE | Homo sapiens | 1/6 |
| Q16584 | hMAP3K11 | TKL | Homo sapiens | 45/44 |
| Q8IW41 | hMAPKAPK5 | CAMK | Homo sapiens | 42/545 |
| P47811 | mp38a | CMGC | Mus musculus | 200/43 |
| XP_341554.1 | rPKCt | AGC | Rattus norvegicus | 157/382 |
| Q02111 | mPKCt | AGC | Mus musculus | 3/1 |
| Q96SB4 | hSRPK1 | CMGC | Homo sapiens | 49/608 |
| Q8IVW4 | hCDKL3 | CMGC | Homo sapiens | 2/5 |
| P42685 | hFRK | TK | Homo sapiens | 172/363 |
| Q60751 | rIGF1R | TK | Rattus norvegicus | 1/0 |
| O95382 | hMAP3K6 | STE | Homo sapiens | 1/6 |
| Q86Z02 | hHIPK1 | CMGC | Homo sapiens | 1/10 |
| Q9Y2K2 | hQSK | CAMK | Homo sapiens | 3/4 |
| P30530 | hAXL | TK | Homo sapiens | 178/321 |
| P52564 | hMKK6 | STE | Homo sapiens | 5/50 |
| P07947 | hYES1 | TK | Homo sapiens | 51/46 |
| Q9H4B4 | hPLK3 | Other | Homo sapiens | 149/727 |
| P06493 | hCDK1 | CMGC | Homo sapiens | 824/1287 |
| Q8IYT8 | hULK2 | Other | Homo sapiens | 2/5 |
| P41241 | mCSK | TK | Mus musculus | 57/28 |
| P20786 | rPDGFRa | TK | Rattus norvegicus | 38/26 |
| Q96PY6 | hNEK1 | Other | Homo sapiens | 3/42 |
| P42681 | hTXK | TK | Homo sapiens | 15/39 |
| Q9NQU5 | hPAK6 | STE | Homo sapiens | 3/39 |
| Q9NY57 | hSTK32B | AGC | Homo sapiens | 2/39 |
| O14757 | hChk1 | CAMK | Homo sapiens | 1024/446 |
| O75914 | hPAK3 | STE | Homo sapiens | 5/57 |
| P31750 | mAKT1 | AGC | Mus musculus | 1/5 |
| Q8N568 | hDCAMKL2 | CAMK | Homo sapiens | 3/40 |
| Q00534 | hCDK6 | CMGC | Homo sapiens | 12/56 |
| Q86UE8 | hTLK2 | Other | Homo sapiens | 5/37 |
| P70618 | rp38a | CMGC | Rattus norvegicus | 45/8 |
| Q9UBE8 | hNLK | CMGC | Homo sapiens | 28/41 |
| P53779 | hJNK3 | CMGC | Homo sapiens | 345/735 |
| Q04771 | hALK2 | TKL | Homo sapiens | 4/37 |
| Q9UIK4 | hDAPK2 | CAMK | Homo sapiens | 6/40 |
| Q9UQM7 | hCAMK2A | CAMK | Homo sapiens | 64/438 |
| Q9NWZ3 | hIRAK4 | TKL | Homo sapiens | 167/738 |
| P36897 | hTGFbR1 | TKL | Homo sapiens | 319/168 |
| Q8NE63 | hHIPK4 | CMGC | Homo sapiens | 160/458 |
| Q92918 | hMAP4K1 | STE | Homo sapiens | 12/29 |
| O15530 | hPDPK1 | AGC | Homo sapiens | 226/782 |
| Q02763 | hTIE2 | TK | Homo sapiens | 417/319 |
| O75385 | hULK1 | Other | Homo sapiens | 2/5 |
| Q13627 | hDYRK1A | CMGC | Homo sapiens | 350/842 |
| Q04912 | hMST1R | TK | Homo sapiens | 112/260 |
| Q08881 | hITK | TK | Homo sapiens | 455/704 |
| Q96RR4 | hCAMKK2 | Other | Homo sapiens | 19/65 |
| O95819 | hMAP4K4 | STE | Homo sapiens | 366/672 |
| P05696 | rPKCa | AGC | Rattus norvegicus | 116/135 |
| Q8MMZ8 | eimPKG | AGC | Eimeria tenella | 256/3 |
| P09769 | hFGR | TK | Homo sapiens | 20/38 |
| P04409 | bPKCa | AGC | Bos taurus | 4/1 |
| P00521 | mlvABL1 | TK | Mus musculus | 2/51 |
| P70336 | rROCK2 | AGC | Rattus norvegicus | 2/1 |
| Q9Y6R4 | hMAP3K4 | STE | Homo sapiens | 2/39 |
| P17612 | hPKAa | AGC | Homo sapiens | 133/582 |
| Q00536 | hPCTK1 | CMGC | Homo sapiens | 13/30 |
| Q99558 | hMAP3K14 | STE | Homo sapiens | 1/9 |
| P16277 | mBLK | TK | Mus musculus | 6/5 |
| Q9C098 | hDCAMKL3 | CAMK | Homo sapiens | 4/37 |
| Q13470 | hTNK1 | TK | Homo sapiens | 11/31 |
| Q03142 | mFGFR4 | TK | Mus musculus | 11/18 |
| Q96RG2 | hPASK | CAMK | Homo sapiens | 16/64 |
| XP_620754.3 | mULK3 | Other | Mus musculus | 4/2 |
| Q9BZL6 | hPKD2 | CAMK | Homo sapiens | 136/663 |
| P46734 | hMKK3 | STE | Homo sapiens | 3/45 |
| P0C264 | hSgK110 | Other | Homo sapiens | 1/6 |
| P35969 | mVGFR1 | TK | Mus musculus | 545/320 |
| Q05397 | hFAK1 | TK | Homo sapiens | 204/806 |
| Q9H2X6 | hHIPK2 | CMGC | Homo sapiens | 208/486 |
| P80192 | hMAP3K9 | TKL | Homo sapiens | 44/47 |
| P22607 | hFGFR3 | TK | Homo sapiens | 42/120 |
| Q9NZJ5 | hPEK | Other | Homo sapiens | 20/17 |
| P29322 | hEPHA8 | TK | Homo sapiens | 10/32 |
| O00444 | hPLK4 | Other | Homo sapiens | 215/557 |
| Q8TD08 | hERK7 | CMGC | Homo sapiens | 10/33 |
| P29597 | hTYK2 | TK | Homo sapiens | 108/657 |
| Q15139 | hPKD1 | CAMK | Homo sapiens | 108/311 |
| P33981 | hMPS1 | Other | Homo sapiens | 9/37 |
| O96017 | hChk2 | CAMK | Homo sapiens | 164/113 |
| Q9WUI1 | mMAPK11 | CMGC | Mus musculus | 129/647 |
| P51813 | hBMX | TK | Homo sapiens | 12/43 |
| P00519 | hABL1 | TK | Homo sapiens | 1128/652 |
| Q99683 | hMAP3K5 | STE | Homo sapiens | 6/55 |
| P36888 | hFLT3 | TK | Homo sapiens | 688/837 |
| Q9H0K1 | hSNF1LK2 | CAMK | Homo sapiens | 27/50 |
| P54646 | hPRKAA2 | CAMK | Homo sapiens | 14/93 |
| Q9Y4K4 | hMAP4K5 | STE | Homo sapiens | 257/457 |
| P48730 | hCSNK1D | CK1 | Homo sapiens | 179/673 |
| Q8N752 | hCSNK1A1L | CK1 | Homo sapiens | 3/35 |
| P42684 | hABL2 | TK | Homo sapiens | 71/108 |
| Q9H3Y6 | hSRMS | TK | Homo sapiens | 48/460 |
| P49185 | rJNK1 | CMGC | Rattus norvegicus | 7/4 |
| P54756 | hEPHA5 | TK | Homo sapiens | 8/36 |
| O00418 | hEEF2K | Other | Homo sapiens | 7/394 |
| Q13873 | hBMPR2 | TKL | Homo sapiens | 3/39 |
| Q9Y2U5 | hMAP3K2 | STE | Homo sapiens | 4/3 |
| Q9H1R3 | hMYLK2 | CAMK | Homo sapiens | 10/32 |
| Q13554 | hCAMK2B | CAMK | Homo sapiens | 61/702 |
| P50613 | hCDK7 | CMGC | Homo sapiens | 158/397 |
| P05480 | mSRC | TK | Mus musculus | 146/554 |
| O76039 | hCDKL5 | CMGC | Homo sapiens | 1/6 |
| Q9Y2H1 | hSTK38L | AGC | Homo sapiens | 3/39 |
| Q15208 | hSTK38 | AGC | Homo sapiens | 2/5 |
| Q9Y616 | hIRAK3 | TKL | Homo sapiens | 6/35 |
| Q13131 | hPRKAA1 | CAMK | Homo sapiens | 166/754 |
| P09216 | rPKCe | AGC | Rattus norvegicus | 101/115 |
| Q13464 | hROCK1 | AGC | Homo sapiens | 597/864 |
| O43353 | hRIPK2 | TKL | Homo sapiens | 14/33 |
| Q9UQ88 | hPITSLREb | CMGC | Homo sapiens | 4/37 |
| Q06187 | hBTK | TK | Homo sapiens | 111/704 |
| P53671 | hLIMK2 | TKL | Homo sapiens | 44/42 |
| P27037 | hACVR2A | TKL | Homo sapiens | 1/40 |
| Q9Y2H9 | hMAST1 | AGC | Homo sapiens | 2/5 |
| Q13153 | hPAK1 | STE | Homo sapiens | 20/727 |
| P42679 | hMATK | TK | Homo sapiens | 31/434 |
| P11309 | hPIM1 | CAMK | Homo sapiens | 406/1137 |
| P54762 | hEPHB1 | TK | Homo sapiens | 8/35 |
| P24941 | hCDK2 | CMGC | Homo sapiens | 1925/2118 |
| P49761 | hCLK3 | CMGC | Homo sapiens | 7/70 |
| Q00537 | hPCTK2 | CMGC | Homo sapiens | 7/35 |
| P21802 | hFGFR2 | TK | Homo sapiens | 70/115 |
| O14976 | hGAK | Other | Homo sapiens | 24/23 |
| Q8NFD2 | hANKK1 | TKL | Homo sapiens | 8/33 |
| Q9P289 | hMST4 | STE | Homo sapiens | 7/44 |
| P49841 | hGSK3b | CMGC | Homo sapiens | 1351/1550 |
| Q08345 | hDDR1 | TK | Homo sapiens | 22/23 |
| O95835 | hLATS1 | AGC | Homo sapiens | 5/36 |
| P51617 | hIRAK1 | TKL | Homo sapiens | 88/518 |
| P00516 | bPRKG1 | AGC | Bos taurus | 118/427 |
| P08631 | hHCK | TK | Homo sapiens | 208/180 |
| P29376 | hLTK | TK | Homo sapiens | 116/271 |
| P25098 | hADRBK1 | AGC | Homo sapiens | 1/7 |
| Q13237 | hPRKG2 | AGC | Homo sapiens | 81/494 |
| P06213 | hINSR | TK | Homo sapiens | 118/488 |
| P49760 | hCLK2 | CMGC | Homo sapiens | 267/713 |
| Q61851 | mFGFR3 | TK | Mus musculus | 116/571 |
| P53350 | hPLK1 | Other | Homo sapiens | 320/998 |
| P41743 | hPKCi | AGC | Homo sapiens | 111/766 |
| Q60737 | mCSNK2A1 | CMGC | Mus musculus | 4/6 |
| Q9P1W9 | hPIM2 | CAMK | Homo sapiens | 205/661 |
| P22612 | hPKAg | AGC | Homo sapiens | 87/490 |
| Q13557 | hCAMK2D | CAMK | Homo sapiens | 166/707 |
| P45983 | hJNK1 | CMGC | Homo sapiens | 512/1226 |
| Q8NEV4 | hMYO3A | STE | Homo sapiens | 3/38 |
| P11275 | rCAMK2A | CAMK | Rattus norvegicus | 4/12 |
| Q9Y5S2 | hCDC42BPB | AGC | Homo sapiens | 3/39 |
| Q99759 | hMAP3K3 | STE | Homo sapiens | 3/5 |
| P06239 | hLCK | TK | Homo sapiens | 1197/1510 |
| Q12851 | hMAP4K2 | STE | Homo sapiens | 250/650 |
| Q96GD4 | hAURb | Other | Homo sapiens | 800/891 |
| Q13546 | hRIPK1 | TKL | Homo sapiens | 5/36 |
| Q14164 | hIKBKE | Other | Homo sapiens | 54/323 |
| P11799 | gMYLK | CAMK | Gallus gallus | 37/96 |
| P49336 | hCDK8 | CMGC | Homo sapiens | 142/325 |
| Q9Y6M4 | hCSNK1G3 | CK1 | Homo sapiens | 65/504 |
| Q64617 | rPKCh | AGC | Rattus norvegicus | 97/113 |
| Q96PF2 | hTSSK2 | CAMK | Homo sapiens | 34/481 |
| Q8TDR2 | hSTK35 | Other | Homo sapiens | 1/6 |
| Q8N5S9 | hCAMKK1 | Other | Homo sapiens | 9/33 |
| Q56UN5 | hYSK4 | STE | Homo sapiens | 7/2 |
| P57059 | hSNF1LK | CAMK | Homo sapiens | 6/36 |
| Q13976 | hPRKG1 | AGC | Homo sapiens | 11/50 |
| O95747 | hOXSR1 | STE | Homo sapiens | 1/6 |
| Q13705 | hACVR2B | TKL | Homo sapiens | 2/41 |
| Q16659 | hERK3 | CMGC | Homo sapiens | 2/40 |
| Q9Y6E0 | hSTK24 | STE | Homo sapiens | 7/35 |
| P15208 | mINSR | TK | Mus musculus | 2/9 |
| Q9JI10 | mSTK3 | STE | Mus musculus | 4/1 |
| Q9P0L2 | hMARK1 | CAMK | Homo sapiens | 5/39 |
| Q05513 | hPKCz | AGC | Homo sapiens | 107/416 |
| P04049 | hRAF1 | TKL | Homo sapiens | 271/154 |
| P27361 | hERK1 | CMGC | Homo sapiens | 84/315 |
| Q9BUB5 | hMKNK1 | CAMK | Homo sapiens | 24/59 |
| Q15835 | hGRK1 | AGC | Homo sapiens | 5/4 |
| Q5VT25 | hCDC42BPA | AGC | Homo sapiens | 86/722 |
| P29320 | hEPHA3 | TK | Homo sapiens | 11/41 |
| P05132 | mPKAa | AGC | Mus musculus | 4/18 |
| Q9HC98 | hNEK6 | Other | Homo sapiens | 2/54 |
| O43283 | hMAP3K13 | TKL | Homo sapiens | 2/5 |
| O00238 | hBMPR1B | TKL | Homo sapiens | 1/6 |
| Q9H2K8 | hTAOK3 | STE | Homo sapiens | 3/6 |
| P51812 | hRSK2 | CAMK | Homo sapiens | 222/887 |
| P15735 | hPHKG2 | CAMK | Homo sapiens | 94/590 |
| P51957 | hNEK4 | Other | Homo sapiens | 68/530 |
| P34947 | hGRK5 | AGC | Homo sapiens | 40/310 |
| Q92630 | hDYRK2 | CMGC | Homo sapiens | 6/7 |
| P14616 | hINSRR | TK | Homo sapiens | 84/688 |
| P49840 | hGSK3a | CMGC | Homo sapiens | 522/1054 |
| P06240 | mLCK | TK | Mus musculus | 49/55 |
| Q14680 | hMELK | CAMK | Homo sapiens | 169/442 |
| P45984 | hJNK2 | CMGC | Homo sapiens | 327/1096 |
| P17252 | hPKCa | AGC | Homo sapiens | 349/739 |
| O15111 | hCHUK | Other | Homo sapiens | 119/509 |
| Q04759 | hPKCt | AGC | Homo sapiens | 425/390 |
| Q9H093 | hNUAK2 | CAMK | Homo sapiens | 11/30 |
| Q09013 | hDMPK | AGC | Homo sapiens | 5/44 |
| P23443 | hRPS6KB1 | AGC | Homo sapiens | 291/568 |
| Q9UHD2 | hTBK1 | Other | Homo sapiens | 47/287 |
| P15056 | hBRAF | TKL | Homo sapiens | 368/175 |
| Q9NRP7 | hSTK36 | Other | Homo sapiens | 5/38 |
| Q8IY84 | hNIM1 | CAMK | Homo sapiens | 1/12 |
| Q2M2I8 | hAAK1 | Other | Homo sapiens | 12/31 |
| P54760 | hEPHB4 | TK | Homo sapiens | 192/277 |
| Q92772 | hCDKL2 | CMGC | Homo sapiens | 2/5 |
| O60285 | hNUAK1 | CAMK | Homo sapiens | 10/55 |
| Q8WXR4 | hMYO3B | STE | Homo sapiens | 3/38 |
| O43781 | hDYRK3 | CMGC | Homo sapiens | 110/418 |
| Q9HBH9 | hMKNK2 | CAMK | Homo sapiens | 171/502 |
| O14965 | hAURa | Other | Homo sapiens | 1007/1150 |
| O00506 | hSTK25 | STE | Homo sapiens | 5/36 |
| O96013 | hPAK4 | STE | Homo sapiens | 111/1036 |
| P18266 | rGSK3b | CMGC | Rattus norvegicus | 5/1 |
| Q9UKE5 | hTNIK | STE | Homo sapiens | 11/32 |
| O75716 | hSTK16 | Other | Homo sapiens | 11/34 |
| O15197 | hEPHB6 | TK | Homo sapiens | 3/4 |
| P07948 | hLYN | TK | Homo sapiens | 364/583 |
| Q8TDC3 | hBRSK1 | CAMK | Homo sapiens | 62/592 |
| P31751 | hAKT2 | AGC | Homo sapiens | 327/906 |
| P51955 | hNEK2 | Other | Homo sapiens | 116/897 |
| P50750 | hCDK9 | CMGC | Homo sapiens | 206/356 |
| P36894 | hBMPR1A | TKL | Homo sapiens | 1/40 |
| P21709 | hEPHA1 | TK | Homo sapiens | 9/36 |
| P04629 | hNTRK1 | TK | Homo sapiens | 286/847 |
| P06494 | rERBB2 | TK | Rattus norvegicus | 64/525 |
| Q96Q40 | hALS2CR7 | CMGC | Homo sapiens | 0/7 |
| P51451 | hBLK | TK | Homo sapiens | 244/463 |
| P45985 | hMKK4 | STE | Homo sapiens | 9/45 |
| P26618 | mPDGFRa | TK | Mus musculus | 142/360 |
| P16092 | mFGFR1 | TK | Mus musculus | 15/31 |
| Q9H2G2 | hSLK | STE | Homo sapiens | 180/482 |
| P12931 | hSRC | TK | Homo sapiens | 1357/1327 |
| P22455 | hFGFR4 | TK | Homo sapiens | 30/111 |
| Q6PHR2 | hULK3 | Other | Homo sapiens | 2/5 |
| P11362 | hFGFR1 | TK | Homo sapiens | 524/1361 |
| Q9UM73 | hALK | TK | Homo sapiens | 220/733 |
| P19784 | hCSNK2A2 | CMGC | Homo sapiens | 33/174 |
| O94806 | hPKD3 | CAMK | Homo sapiens | 294/1008 |
| Q9UBS0 | hRPS6KB2 | AGC | Homo sapiens | 18/30 |
| Q13882 | hPTK6 | TK | Homo sapiens | 44/408 |
| Q01279 | mEGFR | TK | Mus musculus | 7/60 |
| O43293 | hDAPK3 | CAMK | Homo sapiens | 274/744 |
| Q14289 | hFAK2 | TK | Homo sapiens | 105/439 |
| O94768 | hSTK17B | CAMK | Homo sapiens | 6/36 |
| Q7L7X3 | hTAOK1 | STE | Homo sapiens | 127/768 |
| P63319 | rPKCg | AGC | Rattus norvegicus | 194/884 |
| Q9BYT3 | hSTK33 | CAMK | Homo sapiens | 6/43 |
| P09217 | rPKCz | AGC | Rattus norvegicus | 121/790 |
| P53355 | hDAPK1 | CAMK | Homo sapiens | 6/42 |
| P31749 | hAKT1 | AGC | Homo sapiens | 805/1198 |
| O75582 | hMSK1 | AGC | Homo sapiens | 161/448 |
| P52333 | hJAK3 | TK | Homo sapiens | 461/948 |
| Q96NX5 | hCAMK1G | CAMK | Homo sapiens | 29/710 |
| P08069 | hIGF1R | TK | Homo sapiens | 718/1118 |
| P27448 | hMARK3 | CAMK | Homo sapiens | 137/683 |
| Q5S007 | hLRRK2 | TKL | Homo sapiens | 120/219 |
| Q00535 | hCDK5 | CMGC | Homo sapiens | 610/1353 |
| P43403 | hZAP70 | TK | Homo sapiens | 85/149 |
| P00533 | hEGFR | TK | Homo sapiens | 1835/2340 |
| P29323 | hEPHB2 | TK | Homo sapiens | 10/78 |
| Q9UQB9 | hAURc | Other | Homo sapiens | 35/42 |
| Q9BXA7 | hTSSK1 | CAMK | Homo sapiens | 54/506 |
| Q16288 | hNTRK3 | TK | Homo sapiens | 207/528 |
| Q9NYL2 | hZAK | TKL | Homo sapiens | 55/622 |
| P51817 | hPRKX | AGC | Homo sapiens | 205/611 |
| Q00526 | hCDK3 | CMGC | Homo sapiens | 7/76 |
| O00311 | hCDC7 | Other | Homo sapiens | 436/429 |
| P05129 | hPKCg | AGC | Homo sapiens | 209/417 |
| Q16644 | hMAPKAPK3 | CAMK | Homo sapiens | 12/745 |
| Q9H422 | hHIPK3 | CMGC | Homo sapiens | 4/4 |
| P08922 | hROS1 | TK | Homo sapiens | 123/415 |
| Q86V86 | hPIM3 | CAMK | Homo sapiens | 112/551 |
| P07332 | hFES | TK | Homo sapiens | 50/343 |
| P36507 | hMEK2 | STE | Homo sapiens | 14/59 |
| Q96L34 | hMARK4 | CAMK | Homo sapiens | 18/59 |
| P19525 | hEIF2AK2 | Other | Homo sapiens | 5/39 |
| P07333 | hCSF1R | TK | Homo sapiens | 651/823 |
| Q6P3R8 | hNEK5 | Other | Homo sapiens | 1/40 |
| Q9HAZ1 | hCLK4 | CMGC | Homo sapiens | 576/541 |
| P36896 | hALK4 | TKL | Homo sapiens | 74/316 |
| O75676 | hMSK2 | AGC | Homo sapiens | 33/60 |
| P22694 | hPKAb | AGC | Homo sapiens | 93/528 |
| P00523 | gSRC | TK | Gallus gallus | 152/83 |
| P21127 | hPITSLREa | CMGC | Homo sapiens | 3/38 |
| P16056 | mMET | TK | Mus musculus | 130/788 |
| Q86YV6 | hSgK085 | CAMK | Homo sapiens | 6/35 |
| Q9BWU1 | hCDC2L6 | CMGC | Homo sapiens | 21/219 |
| O15075 | hDCAMKL1 | CAMK | Homo sapiens | 17/634 |
| Q14012 | hCAMK1 | CAMK | Homo sapiens | 5/50 |
| Q9P286 | hPAK7 | STE | Homo sapiens | 5/37 |
| P35918 | mVEGFR2 | TK | Mus musculus | 160/52 |
| O14733 | hMKK7 | STE | Homo sapiens | 2/3 |
| Q15746 | hMYLK | CAMK | Homo sapiens | 26/60 |
| O14920 | hIKBKB | Other | Homo sapiens | 453/592 |
| Q02156 | hPKCe | AGC | Homo sapiens | 225/505 |
| P06241 | hFYN | TK | Homo sapiens | 616/404 |
| Q6DT37 | hCDC42BPG | AGC | Homo sapiens | 3/38 |
| P68403 | rPKCb1 | AGC | Rattus norvegicus | 115/134 |
| O00141 | hSGK | AGC | Homo sapiens | 113/746 |
| P16234 | hPDGFRa | TK | Homo sapiens | 293/283 |
| P09619 | hPDGFRb | TK | Homo sapiens | 604/890 |
| Q96KB5 | hPBK | Other | Homo sapiens | 29/558 |
| Q9BQI3 | hHRI | Other | Homo sapiens | 20/8 |
| Q59H18 | hTNNI3K | TKL | Homo sapiens | 7/34 |
| P05771 | hPKCb1 | AGC | Homo sapiens | 295/513 |
| Q8N4C8 | hMINK1 | STE | Homo sapiens | 170/493 |
| Q16566 | hCAMK4 | CAMK | Homo sapiens | 10/64 |
| O75116 | hROCK2 | AGC | Homo sapiens | 716/679 |
| P11440 | mCDC2 | CMGC | Mus musculus | 176/650 |
| Q16512 | hPKN1 | AGC | Homo sapiens | 8/36 |
| Q9Y243 | hAKT3 | AGC | Homo sapiens | 136/902 |
| Q13188 | hSTK3 | STE | Homo sapiens | 213/645 |
| P68400 | hCSNK2A1 | CMGC | Homo sapiens | 176/838 |
| Q07912 | hTNK2 | TK | Homo sapiens | 125/306 |
| P37023 | hALK1 | TKL | Homo sapiens | 2/39 |
| Q8WTQ7 | hGRK7 | AGC | Homo sapiens | 5/3 |
| Q13177 | hPAK2 | STE | Homo sapiens | 3/45 |
| P49187 | rJNK3 | CMGC | Rattus norvegicus | 36/39 |
| O60674 | hJAK2 | TK | Homo sapiens | 524/845 |
| Q16513 | hPKN2 | AGC | Homo sapiens | 186/574 |
| P53778 | hp38g | CMGC | Homo sapiens | 239/216 |
| P54753 | hEPHB3 | TK | Homo sapiens | 17/64 |
| Q9UKI8 | hTLK1 | Other | Homo sapiens | 4/37 |
| Q13164 | hERK5 | CMGC | Homo sapiens | 19/44 |
| P35968 | hVEGFR2 | TK | Homo sapiens | 2556/1563 |
| Q02779 | hMAP3K10 | TKL | Homo sapiens | 23/70 |
| Q13555 | hCAMK2G | CAMK | Homo sapiens | 96/642 |
| Q9NSY1 | hBMP2K | Other | Homo sapiens | 10/32 |
| Q15303 | hHER4 | TK | Homo sapiens | 84/653 |
| Q99640 | hPKMYT1 | Other | Homo sapiens | 1/41 |
| P49137 | hMK2 | CAMK | Homo sapiens | 207/455 |
| P07949 | hRET | TK | Homo sapiens | 338/596 |
| O94804 | hSTK10 | STE | Homo sapiens | 19/25 |
| Q16620 | hNTRK2 | TK | Homo sapiens | 182/810 |
| Q9UPE1 | hMSSK1 | CMGC | Homo sapiens | 3/5 |
| Q7KZI7 | hMARK2 | CAMK | Homo sapiens | 110/597 |
| P00517 | bPKAa | AGC | Bos taurus | 36/142 |
| Q9HCP0 | hCSNK1G1 | CK1 | Homo sapiens | 93/549 |
| Q9UEW8 | hSTK39 | STE | Homo sapiens | 1/6 |
| Q4JIM5 | mABL2 | TK | Mus musculus | 2/2 |
| Q02750 | hMEK1 | STE | Homo sapiens | 322/201 |
| P53667 | hLIMK1 | TKL | Homo sapiens | 132/697 |
| Q9UEE5 | hSTK17A | CAMK | Homo sapiens | 197/435 |
| P49674 | hCSNK1E | CK1 | Homo sapiens | 17/51 |
| Q06418 | hTYRO3 | TK | Homo sapiens | 107/705 |
| P78368 | hCSNK1G2 | CK1 | Homo sapiens | 93/568 |
| P54764 | hEPHA4 | TK | Homo sapiens | 6/41 |
| P63086 | rERK2 | CMGC | Rattus norvegicus | 1/13 |
| P05622 | mPDGFRb | TK | Mus musculus | 163/413 |
| P43405 | hSYK | TK | Homo sapiens | 175/171 |
| Q86UX6 | hSTK32C | AGC | Homo sapiens | 1/40 |
| P30291 | hWEE1 | Other | Homo sapiens | 241/117 |
| Q15831 | hSTK11 | CAMK | Homo sapiens | 6/37 |
| Q15569 | hTESK1 | TKL | Homo sapiens | 3/38 |
| P04626 | hHER2 | TK | Homo sapiens | 735/565 |
| Q9UPZ9 | hICK | CMGC | Homo sapiens | 2/6 |
| P09215 | rPKCd | AGC | Rattus norvegicus | 190/765 |
| P00520 | mABL1 | TK | Mus musculus | 7/5 |
| O14578 | hCIT | AGC | Homo sapiens | 11/31 |
| P49759 | hCLK1 | CMGC | Homo sapiens | 43/48 |
| Q9NYY3 | hPLK2 | Other | Homo sapiens | 24/44 |
| O15264 | hp38d | CMGC | Homo sapiens | 216/182 |
| P10721 | hKIT | TK | Homo sapiens | 553/702 |
| Q13043 | hSTK4 | STE | Homo sapiens | 20/41 |
